# An Assessment of Wound Healing Potential of *Argyreia speciosa* Leaves

**DOI:** 10.1155/2014/406921

**Published:** 2014-01-29

**Authors:** Kuldeep Singh Yadav, Narayan Prasad Yadav, Bindu Rawat, Vineet Kumar Rai, Karuna Shanker, Chandana Venkateswara Rao

**Affiliations:** ^1^Herbal Medicinal Products Department, CSIR-Central Institute of Medicinal and Aromatic Plants, (CIMAP), Lucknow 226 015, India; ^2^Analytical Chemistry Department, CSIR-Central Institute of Medicinal and Aromatic Plants, (CIMAP), Lucknow 226 015, India; ^3^Pharmacognosy and Ethnopharmacology Division, CSIR-National Botanical Research Institute, Rana Pratap Marg, Lucknow 226 001, India

## Abstract

In North India, poultice of young unfolded leaves of *Argyreia speciosa* Linn. (Convolvulaceae) is used for healing wounds. In order to find scientific evidence for the traditional utilization of leaves of *A. speciosa* in wound healing, this investigation was carried out. A linear incision wound of about 3 cm in length and 2 mm in depth and circular excision wound of 177 mm^2^ full thickness were made on the dorsal region of separate groups (*n* = 5) of anesthetized Swiss albino mice. A simple ointment, developed by including ethanol, ethanol-water, and water extracts (10% each, separately) of *A. speciosa*, was applied topically to mice once daily for 14 days after wounding. To evaluate the effect of each extract, wound contraction, epithelization period, wound breaking strength, and hydroxyproline content were determined. The water extract of *A. speciosa* showed accelerated wound healing activity as evidenced by fast wound contraction (96.30 ± 0.52%; *P* < 0.01), rapid epithelization period (11.40 ± 0.60 days; *P* < 0.001), greater wound breaking strength (376.56 ± 21.16 g; *P* < 0.001), and higher hydroxyproline content (16.49 ± 1.12 mg/g; *P* < 0.05) of granulation tissue. The present report supports the traditional use of *Argyreia speciosa* leaves for wound healing and signify its relevant therapeutic potential.

## 1. Introduction

Wound healing is a fundamental response to the tissue injury. It unveils immediately following injury and proceeds in a complicated but well-ordered sequence [1]. The main objective of wound management is to heal the injury in the shortest possible time with minimal pain and discomfort to the patient [2]. Plant derived medicines have been the first line of defense in maintaining health and combating diseases [3]. The Indian subcontinent has a vast repository of medicinal plants that are used in Indian system of medicine [4].

*Argyreia speciosa* Linn. (Convolvulaceae) is widely distributed species in different parts of India. It has been reported to possess nootropic, aphrodisiac, immunomodulatory, hepatoprotective, antioxidant, anti-inflammatory, antihyperglycemic, antidiarrheal, antimicrobial, antiviral, nematocidal, antiulcer, anticonvulsant, analgesic, and central nervous system depressant activities [5]. The leaves of *A. speciosa* are emollient, vesicant, stimulant, and rubefacient and are traditionally used in the treatment of various skin diseases [6, 7]. The roots are beneficial in anaemia, diabetes, obesity, syphilis, tuberculosis, cerebral disorders, and ulcer wound and are also used as aphrodisiac, anti-inflammatory, brain-tonic, cardiotonic, expectorant, digestive, carminative, and appetizer [8].

The seeds of *A. speciosa* principally contain free amino acids, fatty acids, ergometrine, ergoline alkaloids, caffeic acid, and ethyl caffeate. The fruits mostly have n-triacontanol, *β*-sitosterol, p-hydroxycinnamoyl octadecanoate, and caffeic acid. The leaves have rich flavonoid content along with triterpenes, 1-triacontanol, epifriedelinol acetate, epifriedelinol, and *β*-sitosterol. The roots are reported to possess aryl esters, coumarin glycoside, tetradecanoyl palmitate, and 5,8-oxidotetracosan-10-one [5].

Despite the anecdotal evidence of the use of *A. speciosa* as a traditional wound healing agent, the plant has not been explored scientifically to the best of our knowledge. Hence, this study was envisaged to investigate the healing efficacy of *A. speciosa* in experimental full thickness circular excision and linear incision wound model in albino mice.

## 2. Materials and Methods

### 2.1. Collection and Authentication of Plant Material

Fresh leaves of *Argyreia speciosa* was collected in month of October, 2011 from medicinal plant garden of CSIR-CIMAP, Lucknow. The plant was authenticated by taxonomist Dr. S. C. Singh and a herbarium was deposited in botany department of CSIR-CIMAP, Lucknow, and voucher specimen number 13646 was obtained.

### 2.2. Preparation of the Leaves Extract

The fresh leaves were shade dried and coarsely powdered using mechanical grinder. The powdered leaves were macerated in ethanol, ethanol-water (50 : 50, v/v), and water separately for 48 h with occasional shaking. The extracts were concentrated on rotatory evaporator (Rotavapor R-220, Buchi, Switzerland) and dried completely on water bath. The yield of ethanol, ethanol-water, and water extract was found 7.2%, 7.9%, and 9.1% (w/w), respectively. The extracts were stored in closed vials in refrigerator for further use.

### 2.3. Preparation of Topical Formulations

For topical application of the dried extract, the effective concentrations of the extract were determined after scanning a wide concentration range in pilot experiments and the ointments that contained 10% (w/w) dried extract (ethanol, ethanol-water, and water) of *A. speciosa* were made. The ointment base consists of glycol stearate, 1,2-propylene glycol, and liquid paraffin (3 : 6 : 1). This was obtained by melting together with glycol stearate, 1,2-propylene glycol, and liquid paraffin on a hot plate stirrer (RCT Basic, IKA India Pvt. Ltd.) at 45°C. The dried extracts were added to molten base while stirring. The entire mixtures were stirred while cooling until smooth ointments were obtained. The ointments were then filled into 15 g collapsible tubes and marked, respectively.

### 2.4. Experimental Animals

Healthy Swiss albino male mice weighing 20–25 g were used for the experiment. All the animals were acclimatized in group of five in polypropylene cages at controlled environment (20 ± 2°C, 55 ± 5% RH, 12 h dark and light cycle) with free access to standard rodent pellets diet (Dayal Industries, Lucknow) and water *ad libitum*. All experiments were conducted with ethical standards in accordance with Institutional Animal Ethical Committee constituted (Registration no. 400/01/AB/CPCSEA, AH-2012-12) under CPCSEA guidelines.

### 2.5. Experimental Procedure

The animals were randomly allocated to six groups of five mice each in all cases. Group I was kept untreated and considered as negative control while Group II was treated with ointment base only and considered as placebo (vehicle) control. Group III was treated with marketed formulation (1% dry extract of *Centella asiatica*) and served as positive control, while groups IV, V, and VI were treated with 10% w/w plant extract ointments (ethanol, ethanol-water, and water, resp.) and served as test control for both the excision and incision wound model.

#### 2.5.1. Excision Wound Model

The dorsal surface of each animal was shaved by razor and cleaned with 70% ethanol. The animals were left as such for 24 h before creating the wound. A circular impression of 177 mm^2^ was inflicted on shaved dorsal region of each animal. Animals were anaesthetized using intraperitoneal injection of ketamine (90 mg/kg)-xylazine (10 mg/kg) cocktail anesthesia [9] and skin of the impressed area was expurgated with the help of pointed forceps and iris scissor to the full thickness. The animals were then placed into individual cages. A 30 mg each of placebo, marketed formulation, and test ointments were applied topically once a day for 14 days. Percent wound contraction, epithelization period, and hydroxyproline content were estimated after 6 h of last treatment [10].

*Measurement of Wound Contraction and Epithelization Period*. The degree of wound healing was calculated on the basis of continuous scoring method. The wound size was traced on tracing paper every third day and the tracing design was then shifted to graph paper for calculation of surface area of wound considering zero day reading in mm^2^ as 100% [11]. The percent wound contraction was calculated using the following equation:

(1) Wound  Contraction(%) =Initial  wound  size−Specific  day  wound  sizeInitial  wound  size×100.

Falling of scab leaving no raw wound behind was taken as end point of complete epithelization and the days required for this were taken as period of epithelization [11].

*Hydroxyproline Estimation*. Excised tissues were dried in a hot air oven at 60–70°C to constant weight. Dried tissues were hydrolyzed by 6 N HCl at 130°C for 4 h in sealed glass vials. The hydrolysate was neutralized to pH 7.0 and was subjected to Chloramine-T oxidation for 20 min. The reaction was terminated by addition of 0.4 M perchloric acid and color was developed with the help of Ehrlich reagent at 60°C. The absorbance was measured at 557 nm using a spectrophotometer (SpectraMax 190, Molecular Devices, USA). The amount of hydroxyproline in the samples was calculated using a standard curve prepared with pure L-hydroxyproline [12].

#### 2.5.2. Incision Wound Model

Mice were anaesthetized and single full thickness paravertebral 3 cm long incisions were created with a sterile surgical blade and scissors through the skin on the depilated back of mice. After stitching the edges of wound, animals were divided in groups and topically treated with 30 mg each of placebo, marketed formulation, and test ointments, respectively, once a day for the period of 10 days. The wounding day was considered as day 0 (zero). The sutures were removed on the 9th postwounding day when wounds were cured thoroughly and on 10th day, wound breaking strength was measured after 6 h of last treatment [13].

*Measurement of Wound Breaking Strength*. Breaking strength measurement was carried out using CT3 Texture Analyzer (Brookfield Engineering Laboratories, USA) by breaking the skin joint under tension. It indicates how much the repaired tissue resists breaking under tension and may indicate in part the quality of the repaired tissue. It is used to measure the completeness of healing. At 10th day of the study, the animals were sacrificed by cervical dislocation. Mice were hanged on texture analyzer and the breaking strength was noted as peak load in gram using Texture Pro CT V1.3 software [14].

### 2.6. Characterization of Water Extract of *A. speciosa* through HPLC

HPLC analysis of water extract of *A. speciosa* was performed on a system consisting of solvent delivery pumps (LC-20AD), rheodyne manual injector, column oven (CTO-20A), and a multiwavelength photodiode array detector (SPDM20A) linked to a computer system (3000H Series, Lenovo). LC-MS Solution 3.21 (Shimadzu, Japan) was used for equipment control, data acquisition, and processing of the chromatographic information. An ultrasonic bath (Microclean-109, Oscar Ultrasonics, Mumbai, India 30.0 × 25.0 × 12.5 cm, 34 ± 3 kHz, PZT Sandwich type six transducer, 250 W) was used for sonication.

The chromatographic separation was achieved using a Phenomenex C_18_ (4.6 × 250 mm, 5 *μ*m) at 35°C. The mobile phase was 0.5% acetic acid in water (solvent A) and acetonitrile containing 0.5% acetic acid (solvent B). Samples and mobile phase was filtered through a 0.45 *μ*m membrane filter in solvent filtration apparatus (Millipore, USA). The gradient elution was started with 5% B with a flow rate of 1.0 mL/min. The percentage of B was increased to 15% at 10 min and 85% at 45 min. At 50 min, the percentage of B was changed to 95% and at 55 min, this was reduced to 15%. Finally, initial conditions were reverted at 60 min. Detector wavelength was set at 254 nm and the injection volume was 20 *μ*L. The data acquisition was performed in the range of 190–400 nm to monitor any possible coelution in plant sample solution. Therefore, considering maximum chromatographic signal response for fingerprint, wavelength 254 nm was selected. [15].

### 2.7. Statistical Analysis of Data

Data obtained from animal experiments are expressed as the mean with standard error of mean (±SEM). Statistical differences between the treated and the control groups were evaluated by one way ANOVA followed by Tukey's multiple comparison post hoc tests using GraphPad PRISM version 5.01 (GraphPad software, USA). The values of *P* ≤ 0.05 were considered statistically significant.

## 3. Results

### 3.1. Wound Contraction

**Figure 1:**
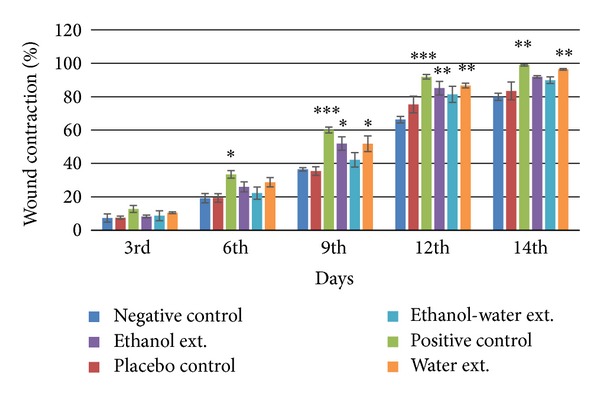
Percent wound contraction in excised wound sites of mice. Values (mean ± SEM) are obtained from each group of five animals (*n* = 5). *P < 0.05, **P < 0.01, and ***P < 0.001 compared to the values of placebo control group. No statistical difference was observed in negative control and placebo control groups.

**Figure 2:**
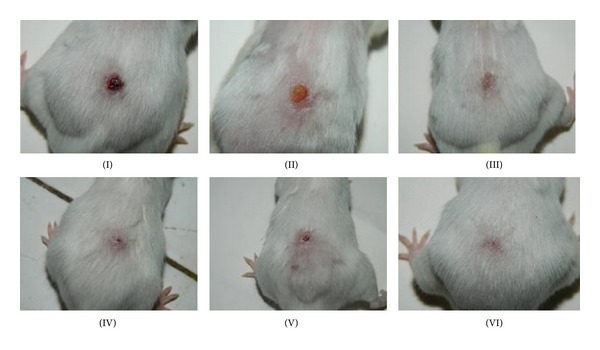
Mice excision wound photographs on 14th postwounding day. Treatment groups: (I) negative control, (II) placebo control, (III) positive control, (IV) ethanol extract, (V) ethanol-water extract, and (VI) water extract.

Mice treated with ethanol extract, ethanol-water extract, and water extract of *A. speciosa* leaves showed 91.85 ± 0.71, 89.81 ± 2.03, and 96.30 ± 0.52% wound closure, respectively, while negative and placebo control groups showed 79.87 ± 2.16% and 83.41 ± 5.35% wound closure, respectively, on 14th postwounding day. The positive control group showed 98.83 ± 0.54% wound closure on last day of wound area measurement. Only water extract and marketed formulation treated groups decreased the wound area significantly (*P* < 0.01) compared to placebo (vehicle) treated group (Figures 1 and 2).

### 3.2. Epithelization Period

**Figure 3:**
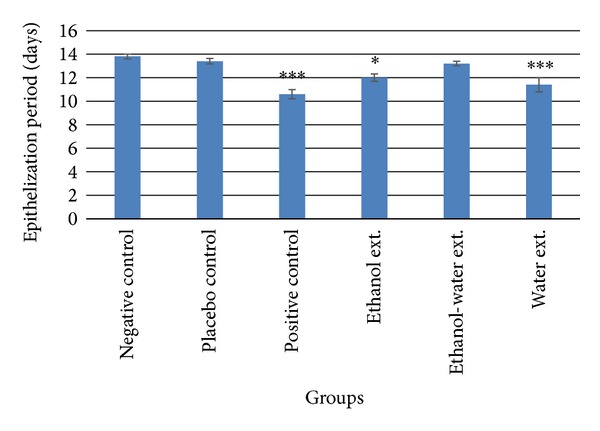
Period of epithelization in skin wound sites. Values (mean ± SEM) are obtained from each group of five animals (*n* = 5). *P < 0.05 and ***P < 0.001 compared to the values of placebo control group. Insignificant difference was perceived in negative control and placebo control groups.

Epithelization period of ethanol extract, ethanol-water extract, and water extract treated group of animals was found to be 12.00 ± 0.32, 13.20 ± 0.20, and 11.40 ± 0.60 days, respectively, whereas negative and placebo control groups displayed epithelization period of 13.80 ± 0.20 and 13.40 ± 0.24 days, respectively. Positive control group exhibited the epithelization period of 10.60 ± 0.40 days. The treatments with marketed formulation, water extract, and ethanol extract were found to be significantly (*P* < 0.05) effective as compared to placebo treatment (Figure 3).

### 3.3. Hydroxyproline Content

**Figure 4:**
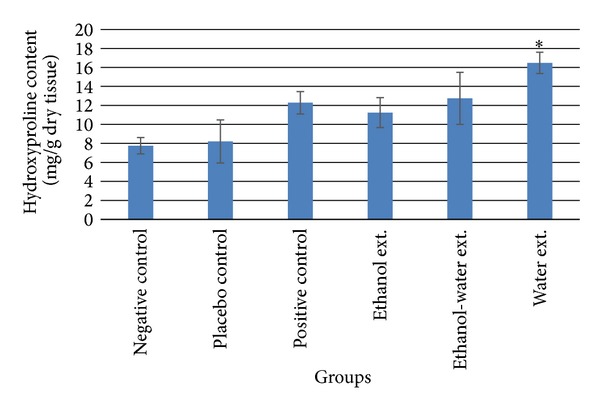
Hydroxyproline content in excised wound tissues. Values (mean ± SEM) are obtained from each group of five animals (*n* = 5). *P < 0.05 compared to the values of placebo control group. Insignificant difference was perceived in negative control and placebo control groups.

The hydroxyproline content of animals treated with ethanol extract was found 11.23 ± 1.58 mg/g dry tissue, ethanol-water extract 12.74 ± 2.74 mg/g, water extract 16.49 ± 1.12 mg/g, negative control group 7.76 ± 0.86 mg/g, and placebo control group 8.21 ± 2.27 mg/g dry tissue, respectively. The hydroxyproline level of positive control group was 12.28 ± 1.18 mg/g dry tissue. The hydroxyproline content of water extract treated group was found to be significantly (*P* < 0.05) higher compared to placebo control (Figure 4).

### 3.4. Wound Breaking Strength

**Figure 5:**
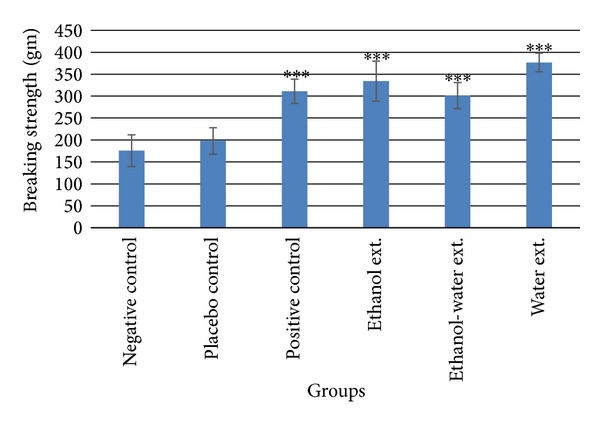
Breaking strength of incised wound skin. Values (mean ± SEM) are obtained from each group of five animals (*n* = 5). ***P < 0.001 compared to the values of placebo control group. No significant difference was obtained in negative control and placebo control groups.

All the extract (ethanol, ethanol-water, and water) as well as marketed formulation treated groups increased the wound breaking strength significantly (*P* < 0.001) compared to placebo control group. The breaking strength of ethanol extract treated group was observed 334.20 ± 45.59 g, ethanol-water extract treated group 301.20 ± 29.75 g, water extract treated group 376.56 ± 21.16 g, and positive control group 310.75 ± 27.59 g, while wound breaking strength of negative and placebo control group was found to be 175.60 ± 36.30 and 198.20 ± 30.14 g, respectively (Figure 5).

## 4. Discussion

Granulation, collagen maturation, and scar formation are some of the many phases of wound healing, which run concurrently, but independent of each other. The use of a single model is inadequate which can collectively represent the various phases of wound healing. Hence, two different models, namely, excision and incision, have been used in the present study to assess the effect of *A. speciosa* on the various phases of wound healing.

Contraction decreases healing time because it decreases the size of the wound and reduces the amount of extracellular matrix needed to repair the defect. Contraction also facilitates reepithelization by shortening the distance for migrating keratinocytes [16]. Moreover, the wound will close at fast rate if the medication is more efficient. In excision wound model, there was an increase in percent wound contraction upon application of the test samples. However, among the three extract studied, the water extract was found to possess better wound contraction rate followed by the ethanolic extract. Hence, it is clear that the water and ethanol extracts of *A. speciosa* are more efficacious than the ethanol-water extract, while the marketed formulation was found to be the most effective among all treatments. The faster wound contraction rate of the aqueous extract may be due to alteration in the function and recruitment of various inflammatory cells, fibroblasts, and keratinocytes [17].

With optimal conditions in the process of wound healing, the epithelization occurs in 48 h. Collagen is required for reepithelization of cell-cell and cell-matrix interactions as it is the key component in hemostasis, which provides strength and integrity to the wound matrix [18]. Among the tested extracts, the epithelization period of water extract treated group was minimum followed by ethanol extract and ethanol-water extract treated group. However, the marketed formulation greatly reduced the period of epithelization and was found more effective than any treatment group. The treatment with water and ethanol extract may contribute to stimulation of cellular proliferation and enhancement of collagen synthesis, thereby facilitating the changes in period of epithelization. As healing phases, namely, inflammation, macrophagia, collagenation, contraction, and epithelization are closely interlinked; it is possible that *A. speciosa* might be influencing the healing process by intervening in one or more phases.

Collagen is the major component of extracellular tissue, which gives strength and support. Breakdown of collagen liberates free hydroxyproline and its peptides. Hence, measurement of hydroxyproline is used as a biochemical marker for tissue collagen and an index for collagen turnover [19]. In the present experiment, increment in hydroxyproline content was observed in all treated groups but the significant difference (*P* ≤ 0.05) was found only in water extract treated group. Increased hydroxyproline content is a reflection of increased collagen content, which implies an effect on the early granulation or proliferation phase in treated wounds.

One of the most crucial phases in dermal wound healing is the progressive increase in biomechanical strength of the tissue; the mechanical properties of the skin are mainly attributed to the function of the dermis in relation to the structure of collagen and elastic fiber networks. Breaking strength of the healed wound is measured as the minimum force required to break the incision apart [20]. Measurement of wound strength provides highly quantifiable estimates of the efficacy of the aggregate healing process [21]. In the present investigation, there was significant (*P* ≤ 0.001) increase in breaking strength in all treated groups. These results suggest that *A. speciosa* has strong wound healing potential. A nonlinear relationship in hydroxyproline levels and wound breaking strength was observed as only water extract treated group increased the hydroxyproline content significantly. It may be due to the difference in mode of action of marketed formulation and *A. speciosa*. These results are similar to the findings of Kaplan et al. [22].

**Figure 6:**
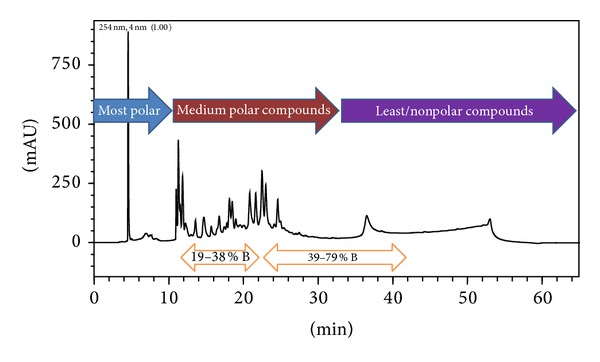
HPLC chromatogram of water extract of *A. speciosa*.

In order to obtain reproducible chromatographic fingerprint of water extract of *A. speciosa* for quality control, the method validation of HPLC-PDA fingerprint analysis was performed on the basis of the retention time and the peak area. The experiment was conducted to examine the classification and concentration of phytochemicals in three categories according to their polarity. Furthermore, due to the lack of reference substances, most of the characteristic peaks could not be identified. However, numbers of flavonoids and alkaloids from this plant have been reported. The possibility of these compounds in medium polar region cannot be ignored. The possible separated chemical flux under experimental conditions, which have chromophoric group, has been shown in the chromatogram. A typical chromatograms of water extract of *A. speciosa* is shown in Figure 6.

## 5. Conclusion

In the present study, ethanol and water extract of *A. speciosa* have shown potential wound healing activity in incision as well as excision wound model in mice. But, water extract was found to be more active as evidenced by significant increase in the rate of wound contraction, breaking strength, hydroxyproline content, and reduced epithelization period. These findings justify inclusion of this plant in the management of wound healing in Indian folk medicine.
